# Ocular Involvement of SARS-CoV-2 in a Polish Cohort of COVID-19-Positive Patients

**DOI:** 10.3390/ijerph18062916

**Published:** 2021-03-12

**Authors:** Joanna Dolar-Szczasny, Mario D. Toro, Anna Dworzańska, Tomasz Wójtowicz, Izabela Korona-Glowniak, Rafał Sawicki, Anastazja Boguszewska, Małgorzata Polz-Dacewicz, Krzysztof Tomasiewicz, Wojciech Załuska, Robert Rejdak, Paola Bagnoli, Dario Rusciano

**Affiliations:** 1 Department of General Ophthalmology, Medical University of Lublin, 20-079 Lublin, Poland; joannaszczasny@op.pl (J.D.-S.); toro.mario@email.it (M.D.T.); robert.rejdak@umlub.pl (R.R.); 2 Faculty of Medical Sciences, Collegium Medicum, Cardinal Stefan Wyszyński University, 01-815 Warsaw, Poland; 3 Department of Infectious Diseases, Medical University of Lublin, 20-059 Lublin, Poland; annadw8@gmail.com (A.D.); tomaskdr@poczta.fm (K.T.); 4 Department of Ophthalmology, Specialist Hospital of Radom, 26-610 Radom, Poland; tomaszpiotrwojtowicz@gmail.com; 5 Department of Pharmaceutical Microbiology, Medical University of Lublin, 20-059 Lublin, Poland; iza.glowniak@umlub.pl; 6 Department of Biochemistry and Biotechnology, Medical University of Lublin, 20-059 Lublin, Poland; rafal.sawicki@umlub.pl; 7 Department of Virology with SARS Laboratory, Medical University of Lublin, 20-059 Lublin, Poland; anastazja.boguszewska@umlub.pl (A.B.); malgorzata.polz-dacewicz@umlub.pl (M.P.-D.); 8 Department of Nephrology, Medical University of Lublin, 20-059 Lublin, Poland; wojciech.zaluska@umlub.pl; 9 Department of Biology, University of Pisa, 56126 Pisa, Italy; paola.bagnoli@unipi.it; 10 Sooft Italia SpA, Research Center, 95123 Catania, Italy

**Keywords:** SARS-CoV-2, COVID-19, eye, lung, PCR

## Abstract

The coronavirus SARS-CoV-2 responsible for the current human COVID-19 pandemic has shown tropism toward different organs with variable efficiency, eyes included. The purpose of this study has been to investigate the presence of detectable SARS-CoV-2 infection in ocular swabs in patients affected by COVID-19. A consecutive series of 74 COVID-19-positive patients (age 21–89) were enrolled at two Polish COVID-19 hospitals for 4 months and were characterized by PCR for the presence of the SARS-CoV-2 genetic material in nasopharyngeal (NP) and ocular swabs, while their respiratory and ocular symptoms were noted. Almost 50% of them presented with severe/critical respiratory involvement, and some degree of eye disease. No tight correlation was observed between the presence of ocular and respiratory symptoms. Three male patients presenting with severe/critical lung disease tested positive in ocular swab, however with mild/moderate ocular symptoms. In conclusion, our study lends further support to the view that overt ocular infection by the SARS-CoV-2 virus is not such a frequent occurrence.

## 1. Introduction

The ophthalmological implications of the severe and acute respiratory coronavirus disease (COVID-19) caused by the strain SARS CoV-2 are not yet well described, and therefore are reason for increasing investigations. The involvement of the eye in the process of SARS CoV-2 infection is suggested by mostly anecdotical evidence on limited numbers of patients, not thoroughly characterized [1]. On the other hand, the implication of ocular infection and the possibility of viral diffusion through the eyes should be seriously taken into consideration [2,3]. Unprotected eyes may be associated with an increased risk of transmission of SARS CoV-2, so that appropriate use of personal protective equipment becomes necessary [4,5]. For this reason, the ongoing COVID-19 pandemic resulted in a temporary halt in all elective ophthalmic surgeries for the protection of patients and healthcare professionals [6,7,8]. On the other hand, with the aging of the population and innovations in treatment, the demand for cataract surgery has been steadily increasing over the past 2 decades, thus addressing the need for critical protective strategies at this time of the COVID-19 pandemic [9].

SARS CoV-2 disease, like other types of viral infection, may be characterized by ocular manifestations (including conjunctivitis), which may present as the initial and the only symptom of infection [10]. Ocular involvement is more likely to be present in severe SARS CoV-2 cases with no age or gender preference. In this respect, the question of whether SARS CoV-2 can be transmitted through the eye should not be ignored. Several properties might render the eye as a potential site for viral infection and dissemination. The eye surface and the nasopharyngeal mucosa are the exposed surfaces amenable to contagion, because they express angiotensin-converting enzyme 2 (ACE2) receptors [11,12]. Moreover, the two compartments are in direct communication through the nasolacrimal duct. Therefore, the eye could be the first entrance door, then diffusing into the nose and throat, or a secondary event, further to the nose infection. In addition, the ocular surface and tear film are potential sites for SARS CoV-2 colonization [13]. In this respect, SARS CoV-2 can survive on the ocular surface longer than in the nasopharynx, with continuous viral replication in the conjunctiva [14]. In addition, SARS CoV-2 can bind to the ACE2 cellular receptor and interact with transmembrane proteases of the host cell, which are expressed in the human cornea, retina, and conjunctival epithelium.

Most importantly, optical coherence tomography has revealed lesions at the level of ganglion cells and inner plexiform layers, indicating neurological damage at the retinal level [15], although the possibility of retinal damage following SARS CoV-2 infection was contradictorily argued in additional correspondence and editorials [16]. More recently, dilated eye examination suggested that patients with severe COVID-19 have acute vascular lesions of the inner retina including flame-shaped hemorrhages and cotton wool spots [17].

Even though existing evidence demonstrates that the eyes are not only an entrance door for the virus, but also a potential source of contagion, the virus appears to be detectable at the ocular level in a small number of positive patients. This might be because SARS CoV-2 detection in the conjunctiva can be difficult, likely because the viral load in the conjunctival sac is relatively low and unstable [18,19,20]. In addition, detection techniques and sampling methodologies appear to differ among different studies, all conditions that might affect viral detection substantially.

Therefore, though several studies, including this one, show a low involvement of the eye, this organ must not be neglected as a potential source of viral diffusion, both inside the subject’s body, and toward neighboring people.

This study wants to provide a further contribution to the understanding of eye involvement in COVID-19 pandemics, describing 74 COVID-19-positive patients sequentially enrolled in a Polish COVID-19 Hospital, and analyzed for their symptoms in the lungs and eyes, and for the presence of the virus in eye swabs.

## 2. Materials and Methods

### 2.1. Patients Recruitment and Nasopharyngeal Swab Collection

This study was conducted on 74 consecutive hospitalized patients who were admitted to the COVID-19 Center at the Department of Infectious Diseases of the Medical University of Lublin, Poland, and at the Specialist Hospital in Radom, Poland, from 22 May to 22 September 2020. Informed consent was obtained from each patient. The research was approved by the Bioethics Committee of the Medical University of Lublin (KE-0254/138/2020). All the enrolled patients tested positive for SARS-CoV-2 according to the WHO interim guidelines. A confirmed case was defined as a positive RT-PCR result in samples from the nasal and pharyngeal swabs. For ethical reasons, no selection was made for untreated patients, so that 32% (24/74) of enrolled patients and 61% (24/39) of those presenting with severe/critical respiratory symptoms, had just started the first dose of an oral antiviral therapy with an association of lopinavir and ritonavir (Kaletra™) [21].

Clinical anamnestic data, like sex, age, comorbidities, drug intake, the occurrence of symptoms respiratory and ocular, recent exposure history, were all obtained directly from patients. All parameters, laboratory findings, and results of imaging tests such as chest computed tomography (CT) and/or X-ray scans were obtained from patients’ electronic medical records. The severity of Covid-19 was established by an assessment of the presence or intensification of respiratory symptoms (cough, dyspnea, shortness of breath) and oxygen saturation level according to the “Management of SARS-CoV-2 infection: recommendations of the Polish Association of Epidemiologists and Infectiologists” [22]. Using these criteria, we then classified enrolled patients into 4 categories: stage 1, stage 2, stage 3 and stage 4 (Table 1).

The staff collecting samples wore complete personnel protective equipment (N95/FFP2 mask, glasses or face shield, apron, and gloves). Dacron-flocked swabs were used for collecting specimens. Patients were asked to position the mask just under the nose to cover the mouth during the procedure. With the participant’s head tilted back slightly, the swab was gently inserted into the nostril along the lateral aspect of the nasal cavity floor and into the nasopharynx. The swab was rotated in place for a few seconds following placement in the nasopharynx and then removed. Swabs were immediately placed in transport medium. After collection, the swabs were transported to the SARS laboratory on ice.

Body temperature was measured immediately before collection of each nasopharyngeal (NP) and ocular swab using a thermo-scan thermometer.

### 2.2. Ocular Swabs Collection and Eye Examination

All patients were questioned about previous ocular surgeries, eye disease, use of eye drops, and use of distance spectacles for refractive errors such as myopia, hyperopia, and astigmatism. All patients were also questioned if they had any ocular symptoms, such as foreign body sensation, ocular pain, burning, eye fatigue, itching or eye discharge.

Any declared symptom was quantitatively assessed 15 min before the ocular swab by an ophthalmologist. A unidimensional visual analog score (VAS) questionnaire 10 cm in length (equivalent to 10 degrees) was used [23]. Before the measurement, the examiner explained to the patient that the 0 points represented no symptom and that the 10 points represented the most intense symptom that he or she felt at that moment. All symptoms were then classified as mild, moderate or severe according to the value declared (none: 0; mild/moderate: from 1 to 7; severe: from 7 to 10.

Then, all patients underwent an ophthalmologic examination to assess any ocular findings. The anterior segment and the ocular surface of both eyes were examined by an experienced ophthalmologist using a portable slit lamp (SL-RVK Portable Hand-Held Slit Lamp, Lunar Health, Irvine, CA, USA). The presence of conjunctivitis was noted and recorded. According to the Efron Grading Scales for Contact Lens Complications, conjunctival hyperemia was classified into five levels of severity—from grade 0 (normal) to grade 4 (severe) and recorded [24]. An indirect binocular ophthalmoscopy was also performed to check any fundus oculi alteration.

Afterwards, conjunctival swabs were collected within three days from the onset of respiratory symptoms to ensure the presence of higher viral load levels to be detected by (RT-PCR) [25,26]. Conjunctival swabs were taken from the inferior conjunctival sac through eversion of the lower eyelids by sterile cotton tips to explore the lower fornixes of the eye [27] without topical anesthesia. Tips of the sterile sticks were placed into liquid Virocult^®^ medium. The personal protective equipment (PPE) used for tear film swab collection and for handling SARS-CoV-2 kit reagents included gloves, eye protection, face mask and lab coats. Sterile gloves were used and changed between patients to avoid contamination risk of both samples and patients.

### 2.3. RNA Extraction and rt-PCR Testing

Both ocular and nasopharyngeal swab specimens were collected in vials containing 1 mL of viral transport medium (VTM, Sigma Virocult^®^, St. Louis, MO, USA) and immediately sent (maintaining proper cold chain) to the Department of Virology within SARS Laboratory, Medical University of Lublin, where they were stored at −40 °C until RT-PCR could be performed. RNAs of 148 samples (two NP swabs per patient) were extracted from VTMs (140 µL per swab) using QIAamp Viral RNA Mini Kit (Qiagen, Hilden, Germany) and eluted in 60 μL. Detection of SARS-CoV-2 was performed using Z-Path-Covid-19-CE Genesig Real-Time RT-PCR kit (Primerdesign, Camberley, UK) according to the manufacturer’s instruction. The quality of the RNA extraction step was verified by amplifying the Genesig^®^ Easy RNA internal extraction control.

Moreover, each sample was also tested with MutaPLEX Coronavirus real time RT-PCR Kit (Immundiagnostik AG) for the simultaneous in vitro detection of RNA of novel coronavirus (SARS-CoV-2) and other beta coronaviruses, extracted from biological specimens. The MutaPLEX^®^ Coronavirus (SARS-CoV-2) real time RT-PCR Kit also contains an Internal System Control (ISC), consisting of primers and probes for the detection of a housekeeping gene (beta-actin, multi-species) in the eluate from a biological specimen. The ISC helps prevent false negative results due to insufficient sample drawing or transport. Bio-Rad CFX Connect™ Real-Time PCR detection system was used to complete all real-time PCRs.

Beta actin control values confirmed that both ocular and nasopharyngeal samples were correctly drawn and transported to the laboratory. Mean ± SD of Ct values for beta-actin gene amplification were found to be 29.38 ± 2.07 (range 24.33–33.63) and 26.20 ± 2.11 (range 22.66–33.48) for ocular and nasopharyngeal samples, respectively (lower Ct values correspond to higher gene load).

### 2.4. Statistic Evaluation

The chi-square test or the Fisher exact test was used to compare groups within tables. A *p* < 0.05 was considered significant.

## 3. Results

Seventy-four patients were enrolled in this study over a 4-month period. This limited number of subjects does not allow a robust statistical analysis; however, it can indicate trends within the different groups identified. Among the 74 patients enrolled in this study, males were predominant over females (46:28), although the mean age was similar: 62 years for males and 60 years for females (Table 2). All patients, by enrollment criteria, tested positive by the NP swab. Two-thirds (56/74) of the patients also tested positive for other beta coronaviruses (beta-CV) by the NP swab, independently from their respiratory symptoms. Table 2 shows the further characteristics of enrolled patients. The body temperature taken at the time of swab collection was higher than 37 °C in the minority of patients: 20 and 19 out of 74, respectively, for NP and ocular swabs. The vast majority (71/74) of patients had detectable respiratory problems at the time of swab collection, with 32 patients with severe lung involvement and in need of oxygen supply, and 6 patients under critical conditions and intubated. Only 3 patients were non-symptomatic. Among the respiratory-impaired patients, 60 showed clear alterations of the lung parenchyma at the CT scan. Roughly half of the patients (35/74) had ocular symptoms, but only 7 showed serious ocular involvement (advanced conjunctivitis). The 3 patients positive for the ocular swab belonged to the mild/moderate ocular symptoms’ cohort. However, 35% (26/74) of the patients showed a positive response in their ocular swabs for other beta-CV, independently from the presence or severity of ocular symptoms and from the positivity of NP swabs: 6/26 were positive for beta-CV in the ocular but not in the NP swab; 2/3 were positive for both SARS-CoV-2 and beta-CV in their ocular swabs. Other beta-CV were detected in 56/74 (75.67%) NP swabs.

Table 3 describes the characteristics of enrolled patients according to sex. Since there is a prevalence of males versus females, the correct comparison must consider the rate of occurrence of the different parameters. Therefore, similar proportions of males and females presented with body temperatures above or below 37 °C at the time of NP or ocular swab collection, although there was a tendency among males towards higher body temperatures. Similarly, the distribution of the gravity of respiratory symptoms and lung involvement by CT scan is similar among males and females, again with a tendency among males towards more severe cases and positive CT scans. Finally, also ocular symptom intensity is equally distributed among males and females, while in this case, more females are affected by severe symptoms, and correspondingly, more males by mild symptoms. Notably, all 3 patients tested positive by the ocular swab were males.

The age distribution of clinical parameters is illustrated in Table 4. Most patients (almost 50%) were over 65 years old, while the minority (15%) were under 40 (Table 4). The trend of age distribution among males and females appeared slightly different, with young subjects more affected among females, and middle-aged subjects more affected among males. Body temperature at the time of swab collection (NP and ocular) was more likely to be higher than 37 °C with increasing age (Table 4): from 9% at ages below 40, to 30% at ages above 65. Respiratory symptoms gravity also tended to increase with increasing age. All patients older than 40 showed evidence of respiratory symptoms, and those with severe/critical conditions increased from 9% among ages below 40, to 61% at ages above 65. Correspondingly, positive TC scans accounted for 36% of patients below 40 and became 82% in patients over 65 (Table 4). Ocular symptoms appeared to be evenly distributed among age classes, at around 50% of incidence. However, severe ocular conditions were more frequent in the intermediate age class (between 40 and 65 years). PCR-positive ocular swabs occurred in one patient of 60 years, one of 75 and one of 80.

**Figure 1:**
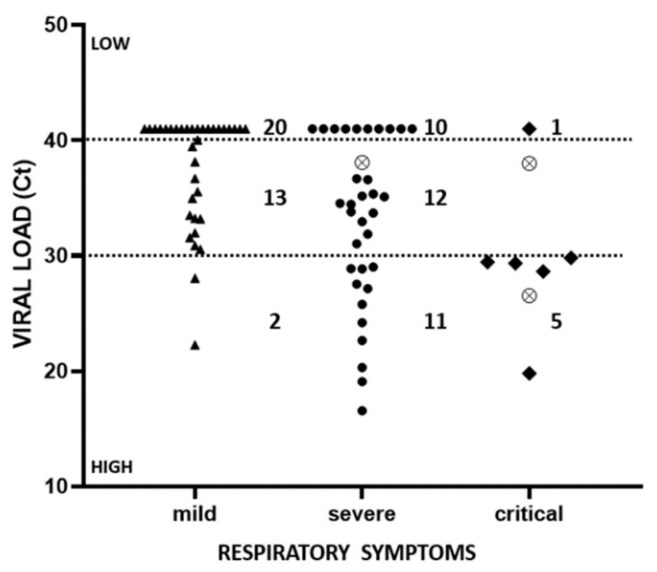
Distribution of patients according to their NP (black symbol) and Ocular (crossed circles) viral load at the moment of hospitalization and the severity of their respiratory symptoms. Values on the ordinate axis report the number of PCR cycles at which the viral RNA became visible. Therefore, the higher this value, the lower the viral load.

Table 5 and Figure 1 show the correspondence between NP or ocular viral load and the gravity of symptoms, respiratory or ocular. Viral load is expressed as the number of real time-PCR cycles (Ct), at which the sample turned positive: the higher this number, the lower the viral load. Ct values higher than 40 mean very low viral load. Low viral loads (Ct > 30) predominate (94%) among patients with mild respiratory symptoms. Patients with severe respiratory symptoms are equally distributed among high, intermediate and low viral loads, while high viral loads dominate (5/6) among patients with critical respiratory symptoms (Table 5). The 3 patients with a positive ocular swab all had body temperature around 37 °C, high NP viral load (Ct < 30) and severe/critical respiratory symptoms, but only mild/moderate ocular symptoms. Their ocular viral load was high in one case, and intermediate in the other two (Table 5).

**Figure 2:**
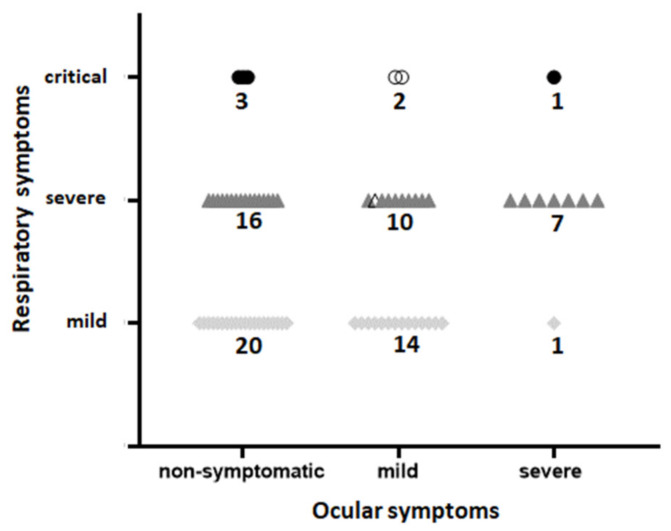
Distribution of patients according to their respiratory and ocular symptoms at the moment of hospitalization. The 3 patients with a positive ocular swab (all with mild ocular symptoms) are identified by empty symbols.

Figure 2 shows the correspondence between respiratory and ocular symptoms. Notably, most of the patients with severe ocular symptoms also had severe/critical (8/9) respiratory symptoms. However, the 39 patients with no ocular symptoms and the 26 patients with mild/moderate ocular symptoms were distributed among the three categories of respiratory symptoms.

## 4. Discussion

The emergence of a new coronavirus strain (SARS-CoV-2) in December 2019 in China led to a pandemic. The lack of herd immunity against this virus and the possibility of viral spread from asymptomatic individuals is still a major challenge for preventing viral transmission (7). The additional finding that the virus can be detected in body secretions other than nasopharyngeal-oropharyngeal swab samples added further complication to the identification of potential sources of viral spread among patients infected with SARS-CoV-2 [28]. In particular, the virus was detected in saliva and in tears, although with a lowest positivity rate [29]. SARS-CoV-2 detection in ocular samples is not unexpected as, for instance, the HIV-1 virus was reported to penetrate various tissues and to exist in bodily fluids and secretions, including tears [30]. In addition, the influenza virus was shown to infect and to replicate at the ocular surface [31].

As for SARS-CoV-2, high viral load in combined nasopharyngeal-oropharyngeal swab samples was associated with high viral load in tears [32], in which the virus could be detected also in asymptomatic patients [33]. Therefore, the potential role of the ocular surface and tears in the viral spread attracted much attention and health experts have warned to avoid touching or rubbing the eyes with dirty hands, to prevent transmission this way [34]. In fact, when sick persons cough or talk, they can release viral droplets from their mouth, right into another person’s face. These droplets, in addition to being inhaled through mucous membranes into the mouth or nose, can also enter through the membranes protecting the eyes—specifically the conjunctiva. This means that the virus can be spread if someone rubs an infected eye and then touches someone else—or even during an eye examination. On the other hand, the viral DNA that can be determined in ocular samples is very low [35] and the question remains on whether the time profile of viral detection may depend on the onset of treatments with antiviral drugs [36]. In our study, 24 patients had started a treatment with Kaletra™ soon before swab collection. This therapy is effective on HIV-1 infections; however, it is scarcely effective on SARS-CoV-2 [21]. Therefore, we consider it unlikely that such treatment may have affected the results here presented. In fact, one of the three patients positive for the ocular swab was under therapy with Kaletra™.

The results obtained in our study shed some more light on the relationship between the respiratory tree and the eye. The two systems are both exposed to the external environment, and to viral aggression from the outside, and are in direct communication via the nasolacrimal duct that drains tears toward the nose and nasopharynx. Although the flux preferentially occurs from the eye to the nose due to the presence of the Hasner valve at the end of the nasolacrimal duct [37], a reflux from the nose to the eye is also possible, as shown by the presence of pepsin in tears of subjects with dry eye and gastroesophageal reflux [38]. Therefore, a viral infection could travel easily from the eye to the nose and the respiratory system, but the inverse path is also possible, although less probable [39].

A meta-analysis considering 11 selected published studies involving 252 COVID-19-positive patients [40] showed that 32% also presented ocular symptoms (it is 47% in our cohort). Only 4.3% (11/252) had PCR detectable SARS-CoV-2 in tears (range 3–16%), not much different from our data, showing 3/74 (4.05%) positive ocular swabs. Another metanalysis [41] reported that conjunctivitis in patients with COVID-19 was more frequent (3%) among those with severe/critical respiratory symptoms than among those with mild respiratory symptoms (0.7%). In our study, we found that 35/74 patients (47.3%) also presented ocular symptoms (Table 3), and that among the 39 patients with severe/critical respiratory symptoms, 20 had also an ocular involvement (51.3%); however, differently from the expectations [42], in only 8 cases, there was a correspondence between severe/critical respiratory symptoms and severe ocular symptoms (8/35 = 22.8%). The remaining 27 patients with ocular symptoms were distributed among different grades of respiratory disease (Figure 2). The three patients with a positive ocular swab all had mild ocular symptoms, with severe/critical respiratory symptoms. Therefore, we did not find a correlation between the grade of respiratory symptoms and the occurrence or the grade of ocular symptoms, likely because we have considered both objective and subjective (VAS questionnaire) evaluations of eye disease. The low incidence of SARS-CoV-2 virus presence in ocular swabs also confirmed in our report (4.05%) may have different explanations. SARS-CoV-2 detection sensitivity in patients’ conjunctiva is below 10% [42], and the number and binding capacity of ACE2 receptors on the ocular surface is lower than in pulmonary tissue [43,44,45]. Therefore, conjunctival swabs are expected to have a lower viral load than NP swabs [46]. The window for virus detection in conjunctival swabs may be no wider than three days [47], although cases with viral persistence of more than two weeks in the conjunctiva have been reported [14,48]. Moreover, tears contain lactoferrin, a natural immunity molecule endowed with antiviral properties [49,50], and secretory IgA [51], which can also be specifically elicited by SARS-CoV-2 infection [52]. Therefore, the low frequency of overt SARS-CoV-2 infections reported in our study can be considered in line with the expectations. A higher frequency has been reported in a recent publication analyzing the presence of SARS-CoV-2 RNA within the cornea of 11 deceased patients, in relation to positive NP (8/11) and conjunctival (5/11) swabs [53]. Four samples showed a positive response both in cornea and conjunctival swab; two samples were positive either in the cornea or in the conjunctival swab, and all the five positive corneas also had a positive NP swab. Such a high frequency of positive ocular findings (6/11) could be due to the selected type of patients, coming from those who succumbed to the viral infection. During our study, we observed the death of 5 patients, all with severe respiratory symptoms, and only one (the 75-year-old male) with a positive ocular swab.

Again, in line with the expectations [54,55] is the finding that not only the number of Covid-positive male patients during the four months of the study was higher than that of females (46:28), but also that severe/critical respiratory symptoms appeared to be slightly more frequent in males (54.4%) than in females (46.4%) (Table 3). On the other hand, the trend for severe conjunctivitis suggested a higher frequency in females (17.9%) than in males (4.4%). Age showed only a weak correlation with the severity of COVID-19 disease [56], and indeed, we found that over the age of 40, more than half of the patients showed severe/critical respiratory symptoms, whereas below 40, it was only 9% (1/11) showing a severe illness (Table 4). No evident correlation between age and eye symptoms was observed, with the exception that middle-aged people (40–65) showed the highest rate (19.2%) of severe conjunctivitis (Table 4). An obvious correlation—although not absolute—was found between viral load and respiratory symptoms [57], in that 89% of the patients (16/18) showing high viral load (Ct < 30) in their NP swab had severe/critical respiratory symptoms, while a lower viral load (Ct > 30) corresponded to both severe and mild respiratory symptoms (Table 5 and Figure 1). A potentially interesting finding is that most of these patients (62/74 = 83.8%) had a concomitant infection by other beta coronaviruses, however with no correlation with ocular or respiratory symptoms. We are not aware of other similar reports, and the finding might suggest, on the one hand, a silent presence of these viruses, and on the other hand, that their presence—if preexisting to the COVID-19 infection—was not protective against the SARS-CoV-2 virus.

Finally, the fact that all three patients with a positive ocular swab were males with a high NP viral load and with severe/critical respiratory symptoms, agrees with all previous findings, and may suggest that the eye infection progressed from the respiratory to the eye, where the viral load appears to be mostly low (Table 5).

## 5. Conclusions

We have reported here the results of 74 consecutive patients characterized for the presence of SARS-CoV-2 in NP and ocular swabs. These results add more knowledge to the limited number of similar studies published so far, in the majority of which an even smaller number of patients (17 to 45) were included [58,59]. In conclusion, and in agreement with these reports, our study lends further support to the view that overt ocular infection by the SARS-CoV-2 virus is not a frequent occurrence, and that there is no evident correlation between eye infection by SARS-CoV-2 and respiratory symptoms.

## Figures and Tables

**Table 1:** Patients’ staging *.

Stage	Oxygen Saturation	Symptoms
1	≥95%	Asymptomatic or paucisymptomatic
2	<95%	Fully symptomatic (mild)
3	<90%	Respiratory failure (severe)
4	ARDS **	ARDS (critical)

* According to: “Management of SARS-CoV-2 infection: recommendations of the Polish Association of Epidemiologists and Infectiologists” 13 October 2020. ** Acute Respiratory Distress Syndrome.

**Table 2:** Patients’ characterization.

**A**			
**SEX**	**N (%)**	**MEAN AGE (Range) ± SD**	
Males	46 (62.2%)	62 (21–89) ± 17.4	
Females	28 (37.8%)	60 (29–87) ± 17.9	
**B**			
**All Patients Positive to the Molecular NasoPharyngeal Swab**			
		***n***	**%**
T °C at NP swab	<37 °C	54	*73*
	>37 °C	20	*27*
T °C at Ocular swab	<37 °C	55	*74.3*
	>37 °C	19	*25.7*
Respiratory Symptoms	none	3	*4.1*
	mild	33	*44.6*
	severe	32	*43.2*
	critical	6	*8.1*
Evidence of lung involvement by CT scan	negative	14	*19*
	positive	60	*81*
Ocular symptoms	none	39	*52.7*
	mild/moderate	28	*37.8*
	severe	7	*9.5*
PCR positive ocular swab		3	*4*

**Table 3:** Patients’ characterization according to sex.

Parameters		Males (46)		Females (28)	
		*n*	%	*n*	%
T °C at NP swab	<37 °C	34	*73.9*	20	*71.4*
	>37 °C	12	*26.1*	8	*21.6*
T °C at Ocular swab	<37 °C	33	*71.7*	22	*78.6*
	>37 °C	13	*28.3*	6	*21.4*
Respiratory Symptoms	none	1	*2.2*	2	*7.1*
	mild	20	*43.5*	13	*46.4*
	severe	21	*45.7*	11	*39.3*
	critical	4	*8.7*	2	*7.1*
Evidence of lung involvement by TC scan	negative	8	*17.4*	7	*25*
	positive	38	*82.6*	21	*75*
Ocular symptoms	none	25	*54.3*	14	*50*
	mild/moderate	19	*41.3*	9	*32.1*
	severe	2	*4.4*	5	*17.9*
PCR positive ocular swab		3	*6.5*	0	*0*

**Table 4:** Patients characterization according to age.

**A**								
**AGE**	***n***	**%**	**Males**	**%**	**Females**		**%**	
<40 years	11	*14.8*	6	13	5		*17.9*	
40–65 years	27	*36.5*	17	37	10		*35.7*	
>65 years	36	*48.7*	23	50	13		*46.4*	
**B**								
**Parameters**		**<40**			**40–65**		**>65**	
			***n***	***%***	***n***	***%***	***n***	***%***
T °C at NP swab		<37 °C	10	90.9	20	74.1	25	69.4
		>37 °C	1	9.1	7	25.9	11	30.6
T °C at Ocular swab		<37 °C	10	90.9	22	81.5	24	66.7
		>37 °C	1	9.1	5	18.5	12	33.3
Respiratory Symptoms		none	3	27.3	0	0	0	0
		mild	7	63.6	12	44.5	14	38.9
		severe	1	9.1	13	48.1	18	50
		critical	0	0	2	7.4	4	11.1
Evidence of lung involvement by TC scan		negative	7	63.6	4	23.5	4	17.4
		positive	4	36.4	13	76.5	19	82.6
Ocular symptoms		none	6	54.5	12	44.5	19	55.9
		mild/moderate	5	45.5	10	37.0	14	41.2
		severe	0	0	5	18.5	1	2.9
PCR positive ocular swab			0	0	1	3.7	2	5.6

**Table 5:** Distribution of patients according to their viral load and ocular or respiratory symptoms.

**A**						
**Viral Load**				**Respiratory Symptoms**	**Viral Load**	**Respiratory Symptoms**
				**Mild**		**Mild**
Ct ≤ 30				2 (5.7%)	11 (33.3%)	5 (83.3%)
30 < Ct ≤ 40				13 (37.15%)	12 (36.4%)	
Ct > 40				20 (57.15%)	10 (30.3%)	1 (16.7%)
Tot. pat.				35	33	6
**B**						
**Age**	**SEX**	**T °C at NP and Ocular Swab**	**NP Swab Viral Load (Ct)**	**Respiratory Symptoms**	**Ocular Swab Viral Load (Ct)**	**Ocular Symptoms**
60	M	36.7	16.56	Severe	38.09	mild/moderate
75	M	36.7	19.8	Critical	26.54	mild/moderate
80	M	37.1	29.45	Critical	38	mild/moderate

## Data Availability

The datasets analyzed in this the study are available from the corresponding author on reasonable request.
